# Therapy of autoinflammatory diseases: a review of the literature

**DOI:** 10.1186/1546-0096-9-S1-P15

**Published:** 2011-09-14

**Authors:** NM ter Haar, J Frenkel

**Affiliations:** 1 Department of Pediatrics University Medical Centre Utrecht, Netherlands

## Background

The evidence for the therapy of autoinflammatory diseases is limited. There are few randomized controlled trials (RCTs) or cohort studies.

## Aim

To provide an up to date literature review about the response to treatment in autoinflammatory diseases.

## Methods

A literature search using Medline and Embase was performed regarding the treatment of Blau’s syndrome, Behçet’s disease, CAPS, CRMO, DIRA, FMF, MKD, NLRP12-mediated periodic fever, PAPA, PFAPA and TRAPS.

## Results

RCTs provide evidence for rilonacept and canakinumab in CAPS, colchicine in FMF and (adeno)tonsillectomy in PFAPA syndrome. For Behçet disease, RCTs have been conducted on the effect of several drugs, including colchicine, azathioprine, cyclosporine A, interferon alfa, etanercept, sucralfate suspension and pimecrolimus. Descriptive studies suggest that NSAIDs and corticosteroids are highly effective in respectively CRMO and PFAPA and moderately effective in the other diseases. Etanercept and anakinra appear to induce a complete or partial response in most patients with MKD, TRAPS, PAPA and colchicine-resistant FMF. Anakinra appears to induce a complete response in the majority of the CAPS and DIRA patients, but seems to be less effective in NLRP12-mediated periodic fever. Complete remission by infliximab is described in cases with Blau’s syndrome, CRMO, PAPA, refractory FMF and Behçet disease, but the effect of infliximab in TRAPS patients seems to be disappointing.

## Conclusion

Reported findings were compared to the results of the Eurofever registry. These combined results could serve as a base for therapeutic guidelines and identify candidate drugs for future therapeutic trials.

